# Supplementary material for: MnTBAP Reverses Pulmonary Vascular Remodeling and Improves Cardiac Function in Experimentally Induced Pulmonary Arterial Hypertension
Source: Int J Mol Sci. 2020 Jun 10;21(11):4130. doi: 10.3390/ijms21114130 (PMC7312610; doi:10.3390/ijms21114130)

Supplementary Materials

Supplementary Figure 1

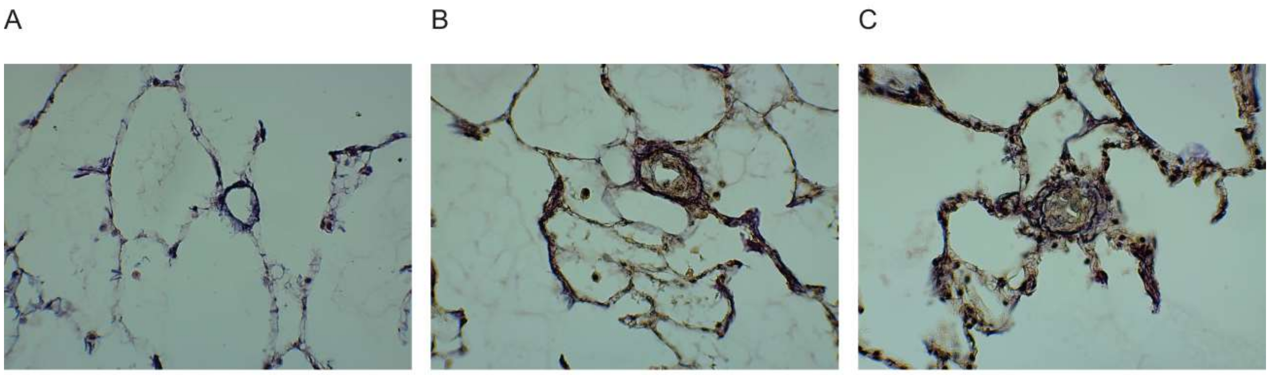

Representative images of an open vessel (A), a partly remodelled vessel (B) and an occluded vessel (C).

Supplementary Figure 2

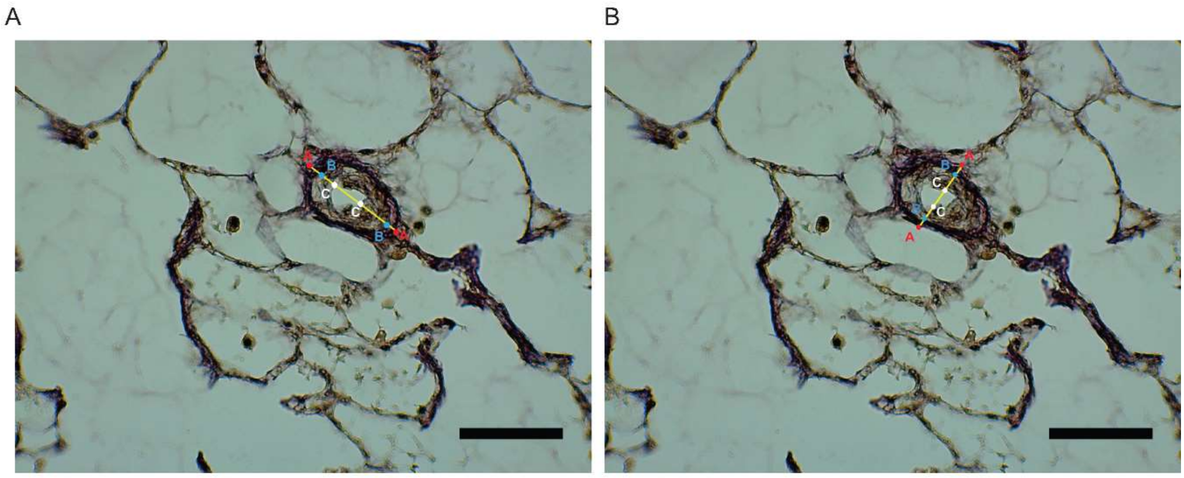

Quantification of pulmonary vascular remodelling.

Intima layer thickness % = [length (BB) – length (CC)] / length (AA)
Media layer thickness % = [length (AA) – length (BB)] / length (AA)

Minimally 20 transversally pulmonary arterioles cut, with an outer diameter between 0 and 30 µm, 30 and 60 µm, 60 and 100 µm, randomly distributed over the lungs, were measured. Every vessel was measured two times as shown by (A) and (B), and the average value was calculated. Scale bar: 50 µm.

Supplementary Figure 3

Vehicle-SuHx

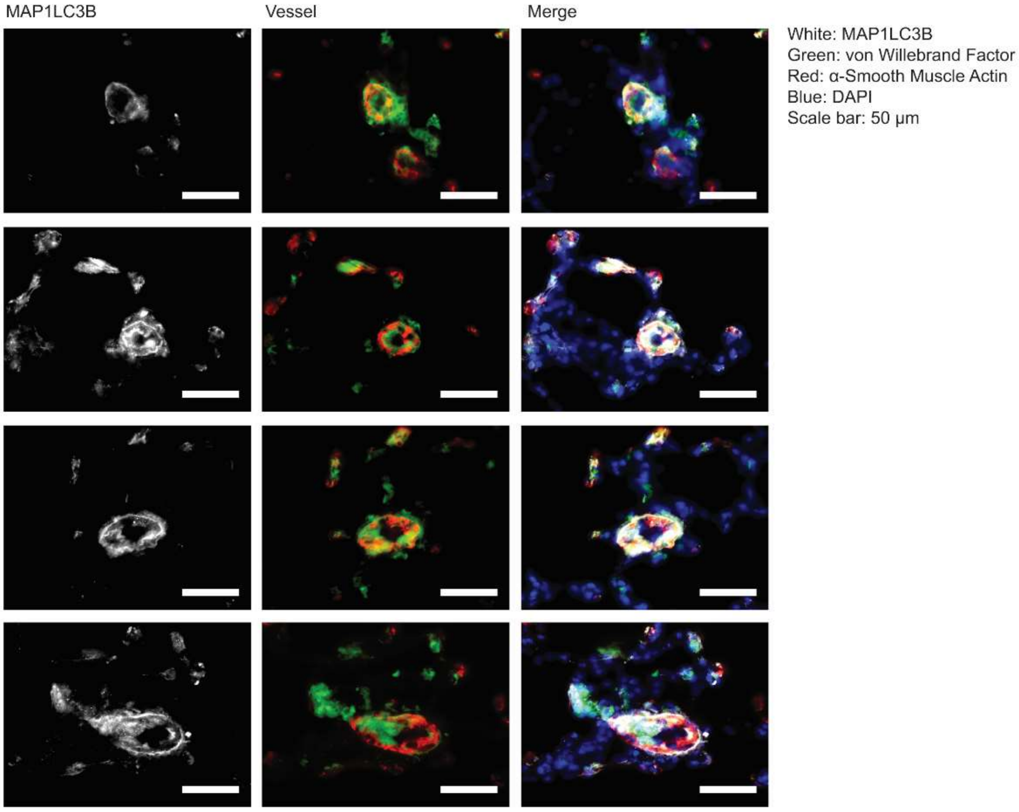

MnTBAP treated

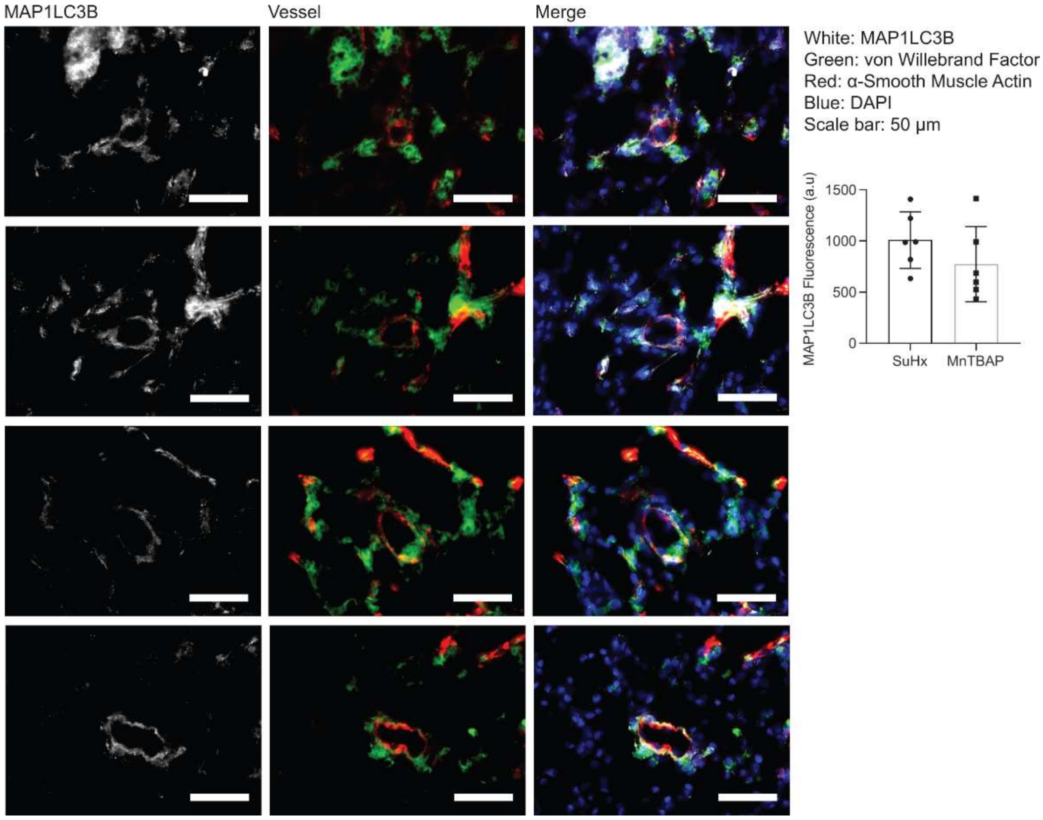

Supplement: Supplementary file 1 [file ijms-21-04130-s001.pdf]
